# Cannabidiol-Loaded Nanostructured Lipid Carriers (NLCs) for Dermal Delivery: Enhancement of Photostability, Cell Viability, and Anti-Inflammatory Activity

**DOI:** 10.3390/pharmaceutics15020537

**Published:** 2023-02-06

**Authors:** Boontida Morakul, Varaporn Buraphacheep Junyaprasert, Krisada Sakchaisri, Veerawat Teeranachaideekul

**Affiliations:** 1 Department of Pharmacy, Faculty of Pharmacy, Mahidol University, Bangkok 10400, Thailand; 2 Department of Pharmacology, Faculty of Pharmacy, Mahidol University, Bangkok 10400, Thailand

**Keywords:** cannabidiol extract, nanostructured lipid carriers, physicochemical properties, permeation, photostability, dermal delivery, anti-inflammatory

## Abstract

The aim of this study was to encapsulate cannabidiol (CBD) extract in nanostructured lipid carriers (NLCs) to improve the chemical stability and anti-inflammatory activity of CBD for dermal delivery. CBD-loaded NLCs (CBD-NLCs) were prepared using cetyl palmitate (CP) as a solid lipid and stabilized with Tego^®^ Care 450 (TG450) or poloxamer 188 (P188) by high-pressure homogenization (HPH). The CBD extract was loaded at 1% *w*/*w*. Three different oils were employed to produce CBD-NLCs, including Transcutol^®^ P, medium-chain triglycerides (MCT), and oleic acid (OA). CBD-NLCs were successfully prepared with an entrapment efficiency (E.E.) of 100%. All formulations showed particle sizes between 160 and 200 nm with PDIs less than 0.10. The type of surfactant and oil used affected the particle sizes, zeta potential, and crystallinity of the CBD-NLCs. CBD-NLCs stabilized with TG450 showed higher crystallinity after production and storage at 30 °C for 30 days as compared to those with P188. Encapsulation of the CBD extract in NLCs enhanced its chemical stability after exposure to simulated sunlight (1000 kJ/m^2^) compared to that of the CBD extract in ethanolic solution. The CBD-NLCs prepared from MCT and OA showed slower CBD release compared with that from Transcutol^®^ P, and the kinetic data for release of CBD from CBD-NLCs followed Higuchi’s release model with a high coefficient of determination (>0.95). The extent of CBD permeation through Strat-M^®^ depended on the oil type. The cytotoxicity of the CBD extract on HaCaT and HDF cells was reduced by encapsulation in the NLCs. The anti-inflammatory activity of the CBD extract in RAW264.7 cell macrophages was enhanced by encapsulation in CBD-NLCs prepared from MCT and OA.

## 1. Introduction

Cannabidiol (CBD) is a nonpsychoactive phytocannabinoid naturally found in *Cannabis sativa* L. During the past decade, Cannabis extracts and CBD in particular have received attention in food, pharmaceutical, and cosmetic applications due to their numerous benefits. Several skin benefits have been reported for Cannabis extracts, including anti-inflammatory, antioxidant, antiaging, and cytoprotective properties without psychoactive effects [1,2]. CBD has been used to treat numerous skin conditions, including urticaria, persistent psoriasis, acne, and epidermolysis bullosa [3,4,5]. As an antioxidant, CBD regulates the redox states of cells by reducing the formation of reactive oxygen species (ROS) and raising the level and activity of both nonenzymatic and enzymatic endogenous antioxidants at the transcriptional level. Additionally, CBD directly protects against functioning antioxidants [6,7]. Recently, Giacoma et al., revealed that hemp water extract containing CBD, cannabidiolic acid, and rutin protected human fibroblasts and keratinocytes from cytotoxicity and apoptosis induced by oxidative stress. In addition, hemp water extracts reduced hydrogen peroxide-induced L-dopa turnover, prostaglandin-E2 production, and the ratio of kynurenine/tryptophane in isolated rat skins, indicating anti-inflammatory and antioxidant effects [6]. Furthermore, CBD treatment of fibroblasts and keratinocytes from psoriatic patients intensified some changes (phospholipid content and membrane charge) caused by radiation and prevented structural and functional changes in healthy skin membranes during phototherapy [2].

CBD is a highly lipophilic molecule with a log P of 6.3 and a molecular weight of 314.46 g/mol [8,9]. Therefore, CBD tends to accumulate in the upper skin layer and permeates poorly into deeper skin layers due to its high lipophilicity [8]. Junaid et al. reported the effects of solvent, drug concentration, chemical enhancer, and essential oil on the percutaneous absorption of CBD [9]. The total amounts of CBD absorbed (CBD in the skin and receptor medium) from 5% (242.41 ± 12.17 µg/cm^2^) and 10% (232.79 ± 20.82 µg/cm^2^) CBD solutions after 24 h were comparable but significantly higher than that of a 1% CBD solution (23.02 ± 4.74 µg/cm^2^). In addition, the enhancement of skin penetration after 4 h was observed when using oleic acid (5% *w*/*w*) as a chemical enhancer but was not found when using Transcutol^®^ P (40% *w*/*w*) and isopropyl myristate (10% *w*/*w*) [9]. Moreover, 5.0% *w*/*w* peppermint oil or eucalyptus oil did not enhance the skin absorption of CBD. In addition, Stinchcomb et al. found that ethanol concentrations between 30 and 33% significantly increased the in vitro human skin transdermal flux of Delta-8-tetrahydrocannabinol and CBD due to an increase in the CBD solubility [10]. CBD can be degraded by light, temperature, and oxidation [11,12]. Therefore, the development of innovative formulations is essential for improving CBD stability and permeability.

Several drug delivery systems have been used to deliver and enhance the stabilities of drugs, such as liposomes, niosomes, ethosomes, polymeric nanoparticles, and lipid nanoparticles [13,14,15,16,17,18]. Lipid nanoparticles, including solid lipid nanoparticles (SLNs) and nanostructured lipid carriers (NLCs), are among the nanoencapsulation materials used to protect and enhance the stabilities of labile drugs [19,20]. They can be used to encapsulate lipophilic, hydrophilic, and amphiphilic drugs. However, particularly high encapsulation efficiencies were reported for lipophilic drugs. The lipid matrices of SLNs are composed of a solid lipid, whereas those of NLCs are prepared from a combination of a solid lipid and oil(s). NLCs have been proven to overcome some drawbacks of SLNs prepared from solid lipids alone, for example, low drug payload, unexpected dynamics of polymorphic transitions, and drug expulsion during storage. The incorporation of oil into the lipid matrix leads to imperfections in the crystals of the lipid matrix, which provides more space for drug accommodation. Previous researchers found that varying the solid lipid, oils, and surfactants altered the characteristics of the lipid nanoparticles [21,22].

Recently, positively charged NLCs containing CBD were prepared by hot microemulsion using stearic acid (1.25%) and OA (0.75%) as the lipid matrix and stabilized with cetylpyridium chloride (0.05%) and Span 20 (0.25%) [23]. An encapsulation efficiency (E.E.) and a drug loading of 99.99% and 18.75%, respectively, were reported. The in vitro release studies showed a biphasic release pattern with a burst release (50% of CBD released in 5 min), followed by slow and sustained release. The cumulative percentages of CBD released from CBD-NLC dispersion and CBD-NLC gel were comparable. The burst release of CBD from NLCs was explained by the enrichment of CBD on the surface layers of the NLCs. However, the physicochemical properties of NLCs also depend on the compositions, such as emulsifier and oil types [19,20,21,22]. Therefore, it was of our interest to investigate the effect of emulsifier (TG450 and P188) and different oils (Transcutol P, MCT, and OA) on the properties and performance of CBD-loaded NLCs.

In this study, we aimed to develop NLCs for topical/dermal applications. Cetyl palmitate (CP) was used as a solid lipid for the CBD-NLCs. To study the effects of formulation compositions on the performance of CBD-NLCs, Tego^®^ Care 450 (TG450) or poloxamer 188 (P188) was used as a surfactant, and three different oils were used to prepare CBD-NLCs, including Transcutol^®^ P, medium chain triglycerides (MCT), and oleic acid (OA). The physicochemical properties of CBD-NLCs were evaluated and compared in terms of particle sizes, size distributions, zeta potentials, crystallinities, encapsulation efficiencies (E.E.), and polymorphism. The stabilities of the CBD extract in the NLCs were evaluated after exposure to simulated sunlight. Furthermore, the in vitro skin performance of CBD was evaluated for release and skin permeation by using Strat-M^®^ as a skin model. Cell cytotoxicity was determined in keratinocytes and fibroblasts, while the anti-inflammatory study was evaluated with lipopolysaccharide (LPS)-induced RAW264.7 cell macrophages.

## 2. Materials and Methods

### 2.1. Materials

Cetyl palmitate (CP) was obtained from SABO S.p.A. (Levate (BG), Italy). Labrafac lipophile WL1340 (medium chain triglycerides, MCT) and Transcutol^®^ P (diethylene glycol monoethyl ether) were gifts from Gattafossé (Cedex, France). Tego^®^ Care 450 (TG450, polyglyceryl-3 methylglucose distearate) was obtained from Evonik Industries AG (Essen, Germany). Poloxamer 188 (P188) was provided by BASF (Ludwigshafen, Germany). Tween 20 and oleic acid (OA) were obtained from Croda (Singapore). CBD extract (87% CBD purity) was purchased from Greenleaf (Bangkok, Thailand). Lipopolysaccharide from Escherichia coli O55:B5 was obtained from Sigma-Aldrich (Missouri, USA). Sodium chloride was acquired from Carlo Erba Reagenti (Cornaredo MI, Italy). Phosphoric acid and potassium dihydrogen orthophosphate were obtained from Fisher Scientific (Loughborough, UK). Methanol was of HPLC grade.

### 2.2. Preparation of CBD-Loaded NLCs

CP was used as a solid lipid matrix, and TG450 or P188 was used as surfactants. The addition of Transcutol^®^ P, MCT, or OA as an oil into a solid matrix was performed to prepare the CBD-NLCs. All lipid nanoparticles were produced by the hot high-pressure homogenization technique (HPH) [24]. In the preparation, the lipid phase containing a solid lipid, oil, and CBD extract (1.0% *w*/*w*) was melted at between 75 °C and 80 °C. Then, the hot aqueous surfactant solution (TG450 or P188) at 80–85 °C was added to the melted lipid phase under stirring by an Ultra-Turrax^®^ T25 (IKA, Staufen, Germany) at 8000 rpm for 1 min. Subsequently, the pre-emulsion was then placed in the high-pressure homogenizer (APV Gaulin, Lübeck, Germany) for three cycles at 500 bar. The hot nanoemulsions were subsequently cooled to room temperature under ambient conditions, generating lipid nanoparticles. Finally, Unigerm G2 was added to the CBD-NLC dispersions to preserve formulations from microorganisms. The compositions of all prepared formulations are shown in Table 1.

### 2.3. Particle Sizes and Size Distributions

The mean particle size and the particle size distribution (polydispersity index, PDI) were determined by photon correlation spectroscopy (PCS) using a Zetasizer NanoZS (Malvern Instruments, Worcestershire, UK). The sample was diluted with sterile water for injection to obtain a suitable scattering intensity. The mean particle size and PDI were obtained by averaging the values from three measurements at an angle of 173° in a 10 mm diameter cell at 25 °C.

### 2.4. Zeta Potential

The zeta potential (ZP), also known as the electrokinetic potential, is defined as the value of the electrical potential at the shear plane of a particle and indicates the physical stability of a colloidal system. The ZP was determined with a Malvern Zetasizer NanoZS by averaging the results of three measurements performed at 25 °C. The ZP values were calculated using the Helmholtz–Smoluchowski equation. To reduce the fluctuations in water conductivity between days, all samples were diluted with distilled water and adjusted the conductivity to 50 S/cm with a 0.9% sodium chloride solution before the measurement.

### 2.5. Particle Morphology

The particle morphologies of CBD-NLCs were examined with a transmission electron microscope (TEM) (Hitachi model HT-7700, Japan). One drop of CBD-NLCs was deposited on a copper grid after being diluted with filtered water and negatively stained with 1% uranyl acetate. The test samples were air-dried at room temperature for one hour. The CBD-NLC grids were stored in a desiccator until they were used.

### 2.6. Drug Encapsulation Efficiency (E.E.)

To determine the amount of CBD entrapped in NLCs, the ultrafiltration technique was used to separate CBD in lipid nanoparticles and free CBD in the filtrate. Briefly, approximately 1 g of lipid nanoparticles was added onto the filter membrane of the ultracentrifuge tube with a molecular weight cutoff of 50 kDa (Amicon^®^ Ultra15 centrifugal filter, Merck KGaA, Darmstadt, Germany). Then, the separation of free CBD and CBD entrapped in lipid nanoparticles was performed by centrifugation at 4000 rpm for 60 min with a Centrifuge Model 5430 (Eppendorf, Germany). Afterward, the free CBD in the filtrate was analyzed by high-performance liquid chromatography (HPLC) using a Shimadzu LC-20AD system (Shimadzu, Japan). The stationary phase was Platisil ODS (5 µm, 250 mm × 4.6 mm, Dikma, CA, USA). The mobile phase consisted of methanol and water (85:15 *v*/*v*). The flow rate of mobile phase was 1.2 mL/min. The wavelength for detection was set at 220 nm. The injection volume of the standard and test samples was 20 µL. The E.E. of CBD in NLCs was calculated with Equation (1).

(1) $$\%E.E.=\frac{\left(\mathrm{Total}\;\mathrm{amount}\;\mathrm{of}\;\mathrm{CBD}\;-\;\mathrm{Free}\;\mathrm{amount}\;\mathrm{of}\;\mathrm{CBD}\right)\times 100}{\mathrm{Total}\;\mathrm{amount}\;\mathrm{of}\;\mathrm{CBD}}$$

### 2.7. Thermal Analysis

The thermal behaviors of CP, CBD extract, and CBD-NLCs were analyzed with a Mettler DSC1 apparatus (Mettler Toledo, Switzerland). CBD-NLCs samples weighing approximately 10–20 mg (related to 1–2 mg of lipid content) were placed into a 40 µL aluminum pan and covered with an aluminum pan lid. The sample was heated from 20 °C to 85 °C and then cooled to 20 °C with a heating/cooling rate of 5 °C/min and a nitrogen flushing rate of 80 mL/min. An empty aluminum pan covered with an aluminum pan lid was employed as a reference. The melting temperatures (T_m_), melting enthalpies (∆H), and onset temperatures (T_onset_) were analyzed using STAR^e^ Software version 16.10. The degree of lipid crystallinity was calculated as the percentage of CI (%CI) according to Equation (2):

(2) $$\%\mathrm{CI}\;=\frac{\left(\Delta H_{\mathrm{lipid}\;\mathrm{nanoparticles}}\right)}{\Delta H_{\mathrm{bulk}\;\mathrm{lipid}}\times \%\mathrm{lipid}\;\mathrm{phase}}\times 100\;$$

### 2.8. X-ray Diffraction (XRD)

The polymorphism forms of the lipid matrix were also assessed by utilizing a wide-angle X-ray diffractometer (Miniflex 600, Rigaku, The Woodlands, TX, USA) with a copper anode (Cu-K_α_ radiation, 40 kV, 15 mA, λ = 1.54056 A°) and a goniometer as a detector. The test samples (i.e., bulk CP, CBD extract, and CBD-NLCs) were set up on a quartz sample holder. The CBD-NLCs were mixed with locust bean gum to form a paste before the measurements. The experiments were performed with a scan rate of 0.02°/min within the diffraction angle range.

### 2.9. Photostabilities of CBD-NLCs

The stabilities of CBD entrapped in NLCs were evaluated by exposure to simulated direct sunlight. The samples were kept in clear glass vials. The photostabilities were determined using a Q-Sun Xe-1 Xenon Test Chamber connected to a cooling system (Q-Lab Corporation, Westlake, OH, USA). The Daylight-BB optical filter was used to generate outdoor sunlight. The temperature was controlled at 30–35 °C. The CBD remaining in the NLCs was analyzed after exposure to 100, 300, 500, 700, and 1000 kJ/m^2^ of simulated sunlight. To determine the amount of CBD remaining in NLCs, the CBD-NLC dispersion after exposure to simulated light was added to a volumetric flask, then methanol was added and sonicated for 15 min to extract CBD from NLCs. After cooling down to room temperature, the solution was filtered through a 0.22 μm cellulose acetate syringe membrane filter (Filtrex, Milano, Italy) and analyzed by HPLC as previously described. One percent CBD extract in absolute ethanol was used as a reference for comparison.

### 2.10. In Vitro Release Study

In vitro release studies of CBD-NLCs were performed using static Franz diffusion cells (7 mL, surface area 0.60 cm^2^, DHC-6T, Logan Instruments Corporation, New Jersey, NJ, USA). A polycarbonate membrane filter with pore sizes of 0.05 µm (SterliTech, Washington, DC, USA) was mounted between the donor and receptor compartments. The temperature was controlled at 32 ± 0.3 °C. The receptor medium was a 2% Tween 20 solution (pH~5.0–5.5), and it was continuously stirred with a magnetic stirrer at 500 rpm. The 300 µL test samples were added to the donor compartment. At each time point (2, 4, 6, 8, 10, 12, and 24 h), 500 µL of the receptor medium was collected and replaced with fresh receptor medium. As previously stated, the quantity of CBD in the receptor phase was determined using HPLC. The experiments were performed in triplicate for each formulation.

To evaluate the release kinetics of the CBD-NLCs, the cumulative amount of CBD per area (µg/cm^2^) in the receptor phase was plotted as a function of time and fitted to kinetic models, including zero-order kinetics, first-order kinetics, Higuchi’s kinetics, and Korsmeyer–Peppas kinetics [25], as described in Equations (3)–(6):

Zero-order M_t_ = M_0_ + kt (3)

First-order M_t_ = M_α_ (1 − e^−kt^) (4)

Higuchi’s model M_t_ = M_α_ + kt^(1/2)^ (5)

Korsmeyer-Peppas M_t_/M_α_ = kt^n^ (6)

where M_t_ and M_0_ are the amounts of drug released at time t and at time = 0, respectively, k is the release rate constant, M_t_/M_α_ is the fraction of drug released, and n is the diffusional release exponent, which is indicative of the release mechanism.

### 2.11. In Vitro Skin Permeation Study

In vitro skin permeation studies of CBD-NLCs were conducted with static Franz diffusion cells (7 mL, surface area 0.60 cm^2^, DHC-6T, Logan Instruments Corporation, Somerset, NJ, USA) with a Strat-M^®^ membrane (EMD Millipore, Burlington, MA, USA) mounted between the donor and receptor compartments, with the shiny side of the Strat-M^®^ membrane in contact with the donor compartment [26]. The temperature was controlled at 37 ± 0.3 °C. The receptor compartment was filled with 2% Tween 20 in a phosphate buffer (pH~7.4) and stirred with a magnetic stirrer at 500 rpm. The membrane was equilibrated at 37 ± 0.3 °C for 30 min before the test samples (300 µL) were added to the donor compartment. At predefined intervals (2, 4, 6, 8, 10, 12, and 24 h), 500 µL of the receptor medium was withdrawn and replaced with new receptor medium. The amount of CBD in the receptor phase was measured using HPLC. The experiments for each formulation were performed in triplicate. The cumulative amount of CBD permeated through Strat-M^®^ membrane from each formulation was compared.

### 2.12. Cytotoxicity in Skin Cells

To evaluate the cytotoxicities of the CBD extract and CBD-NLCs, the test samples were assessed using mitochondrial reduction of [3-(4,5-dimethylthiazol-2-yl)-5-(3-carboxymethoxyphenyl)-2-(4-sulfophenyl)-2H-tetrazolium] or MTS (CellTiter 96^®^ AQueous One Solution Reagent, Promega, Fitchburg, WI, USA) in the human foreskin fibroblast cell line (HDF) (ATCC^®^ PCS-201-012) and the human immortalized keratinocyte cell line (HaCaT) (CLS Cell Lines Service GmbH, Germany).

#### 2.12.1. Cell Preparation

First, the cells were cultured in a 75 cm^3^ tissue culture flask using culture media containing 10% FBS and 90% DMEM with 1 mM sodium pyruvate, 100 U/mL penicillin G, and 100 g/mL streptomycin. The culture media was replaced every 48 h until the cells were at approximately 80% confluence in a humidified incubator (5% CO_2_ at 37 °C). Then, a 0.05% trypsin/EDTA solution was used to trypsinize the cells. Subsequently, approximately 10,000 fibroblast cells or 8000 keratinocyte cells were seeded in each well of a flat transparent 96-well plate at a volume of 100 µL per well.

#### 2.12.2. MTS Cell Proliferation Assay

The cytotoxicities were determined with the MTS assay, as previously described by Wang et al. [27] and Riebeling et al. [28] with some modifications. After seeding for 24 h, the well-plates were treated with different concentrations of samples. The cells treated with the culture medium were used as a control (untreated). After 24 h of incubation, the supernatant was removed, and the cells gently rinsed with PBS (0.01 mM, pH 7.4) twice. Then, 120 µL of culture medium containing MTS solution at a ratio of 5:1 (*v*/*v*) was added and incubated (5% CO_2_ at 37 °C) for 3 h. After 3 h of incubation, the formation of soluble formazan was evaluated by the colorimetric method at 490 nm using a microplate reader (CLARIOstar Plus, BMG Labtech, Ortenberg, Germany). Finally, cell viabilities after the different treatments were calculated by comparing the treated cells to the untreated cells as in Equation (7):

(7) $$\%\mathrm{Cell}\;\mathrm{viability}=\frac{A}{B}\times 100$$

where A and B are the absorbance of treated and untreated samples, respectively.

### 2.13. Anti-Inflammatory Study

#### 2.13.1. Cell Culture Preparation

The anti-inflammatory activities of the CBD extract and CBD-NLCs were evaluated in RAW264.7 cell macrophages (ATCC TIB-71TM) by determining the in vitro production of interleukin-6 (IL-6). RAW 264.7 cell macrophages were cultured in DMEM supplemented with 10% FBS, 1% NEAAs, and 1% penicillin/streptomycin (P/S). Cells were incubated at 37 °C in 5% CO_2_ until 80% confluence was observed.

#### 2.13.2. Cell Viability

A 3-(4,5-dimethylthiazol-2-yl)-2,5-diphenyl tetrazolium bromide (MTT) assay was used to determine cell viability. In brief, RAW264.7 cell macrophages were seeded in a 96-well, flat-bottom cell culture plate at a concentration of 1 × 10^4^ cell/well in 180 µL of cell culture medium, and the cells were incubated overnight at 37 °C under 5% CO_2_. Afterward, the cells were treated with the test samples in triplicate at different concentrations for 24 h. Then, the culture medium was removed and replaced with 100 µL of MTT solution. After 30 min of incubation, the MTT solution was removed by aspiration. The insoluble formazan in RAW 264.7 cell macrophages was extracted using 180 µL of DMSO, and the optical density (OD) was detected at 570 nm with a microplate reader. The cell viability was computed according to Equation (7).

#### 2.13.3. Measurement of IL-6 Production

CBD extract and CBD-NLCs with concentrations showing cell viabilities of higher than 80% were incubated with RAW264.7 cell macrophages as previously described by Teeranachaideekul et al. [29] with some modifications. The cells were plated in a 24-well plate at a concentration of 1 × 10^5^ cells/well and incubated at 37 °C under 5% CO_2_ for 24 h. After that, the culture medium was removed and replaced with fresh culture medium containing test samples for 2 h. Then, 1 µg/mL lipopolysaccharide (LPS) was added to each well for 24 h of incubation at 37 °C under 5% CO_2_. RAW264.7 cell macrophages treated with and without LPS were used as positive and negative controls, respectively. Diclofenac sodium at the concentration of 1000 μM was used as a standard anti-inflammatory agent [29]. The concentration of IL-6 in culture media was determined using ELISA MAX^TM^ Standard Set Mouse IL-6 (Biolegend, California, USA) according to the manufacturer’s instructions in a 96-well plate.

### 2.14. Statistical Analysis

All data are expressed as the mean ± standard deviation. Statistical analyses were performed using SPSS version 18. One-way ANOVA was applied to determine significant differences among samples. The *p*-value for a significant difference was set at less than or equal to 0.05 (*p* < 0.05).

## 3. Results and Discussions

### 3.1. Particle Size and Size Distribution Analysis

Table 1 shows the compositions of the CBD-NLC formulations. The lipid matrix for the CBD-NLCs comprised CP and oil at a ratio of 8:2. The CBD extract was loaded at a concentration of 1% *w*/*w*, corresponding to 0.87% CBD. TG450 (1.8% *w*/*w*) or P188 (2.5% *w*/*w*) was used as a stabilizer. Unigerm G2 (1% *w*/*w*) was used as a preservative agent.

After production, the particle sizes of the CBD-NLCs were in the range 163–176 nm with a PDI value lower than 0.1, as shown in Table 2. All formulations showed PDI values of less than 0.1, indicating narrow size distributions [30]. The obtained results suggested that HPH was a suitable technique for preparing CBD-NLCs.

### 3.2. Zeta Potential

The zeta potential values of CBD-NLCs were between |−29 mV| and |−57 mV| depending on the type of surfactant and oil composition used, as depicted in Table 2. CBD-NLC samples stabilized with TG450 (CBD-NLC1) exhibited higher zeta potential values (|−57 mV|) than those stabilized with P188 (between |−29 mV| and |−40 mV|). The high ZP values of CBD-NLC1 stabilized with TG450 were attributed to dissociation of a fatty acid (stearic acid), which is a hydrophobic part of TG450, generating a highly negative charge at the surface of CBD-NLCs [24]. For CBD-NLC2, CBD-NLC3, and CBD-NLC4, they were stabilized with the same emulsifier (i.e., P188); however, the zeta potential of CBD-NLC4 was higher than that of CBD-NLC2 and CBD-NLC3 (*p* < 0.05). The higher negative value for CBD-NLC4 was likely due to the ionized fatty acids of OA [31]. As shown in Table 2, the zeta potentials of all formulations were almost equal to or higher than |−30 mV|, implying that all CBD-NLC formulations were physically stable.

### 3.3. Particle Morphology

**Figure 1:**
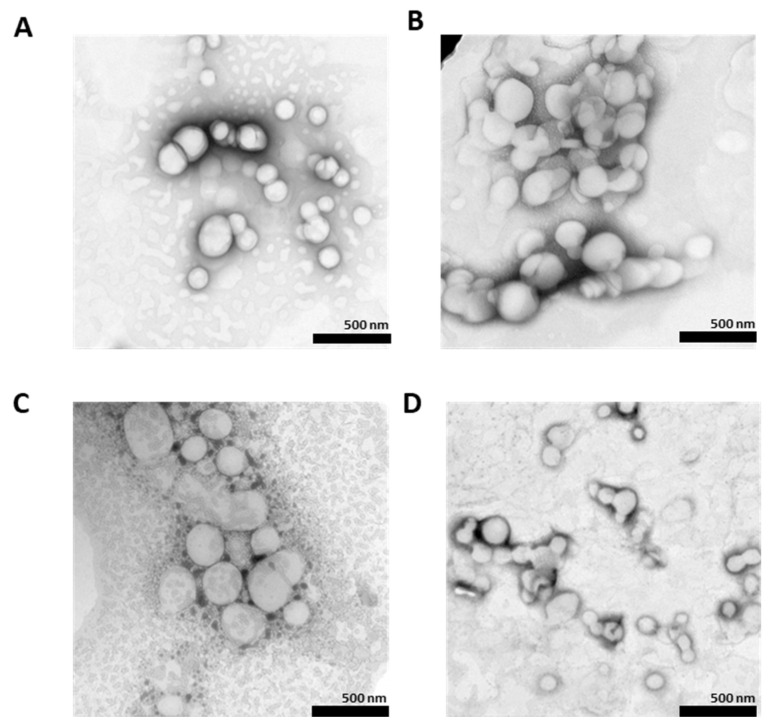
Negative staining TEM images of (**A**) (CBD-NLC1), (**B**) (CBD-NLC2), (**C**) (CBD-NLC3), and (**D**) (CBD-NLC4) at ×10,000 magnification.

Figure 1 shows TEM images of CBD-NLCs stained with 1.0% uranyl acid. The CBD-NLCs formed spherical particles. The TEM images revealed that the particle sizes of the CBD-NLCs were between 100 and 300 nm, which is in accordance with the data from particle size analysis with the Zetasizer NanoZS system.

### 3.4. Encapsulation Efficiency

**Figure 2:**
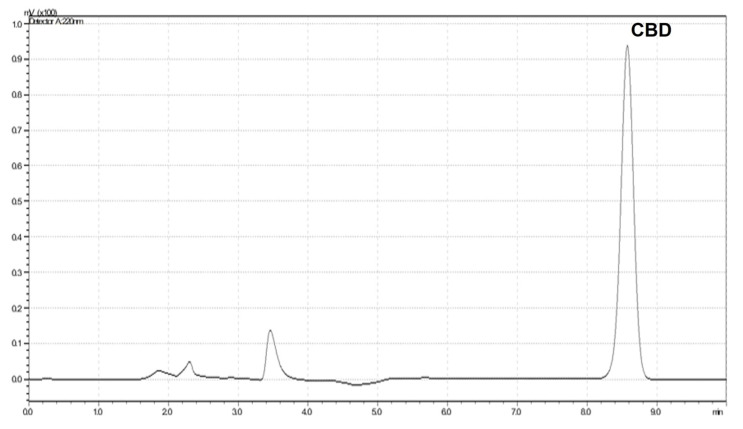
HPLC chromatogram of CBD.

Quantitative analysis of CBD contents in the formulation and the filtrate was investigated via HPLC with a UV detector. The retention time for CBD was between 8.6 and 8.7 min, as shown in Figure 2. A calibration curve was prepared in the range 1.25–40 µg/mL with a correlation coefficient (r) of greater than 0.99. The relative standard deviations for intraday and interday precision were less than 2%. The recovery was in the range of 90–110%.

The amounts of CBD in the filtrates of all CBD-NLCs were analyzed. No CBD peak was detected in the HPLC chromatogram. Therefore, the E.E. was assumed to be 100%. Moreover, no CBD crystals were observed with a polarized light microscope. The high E.E.s for the CBD in lipid nanoparticles were likely due to the high lipophilicity of CBD (log P = 6.3). As a result, CBD was easily dissolved in the lipid matrices of the NLCs. Similarly, several other studies reported high E.E. (almost 100%) for CBD in nanocarriers such as nanoemulsions, microemulsions, nanocapsules, and NLCs [32,33,34,35]. The above results showed that NLCs are suitable nanocarriers for the encapsulation of CBD.

### 3.5. Thermal Analysis

Thermal analyses were performed to evaluate the onset temperatures, melting temperatures, and lipid crystallinities (%CI) of bulk CP, CBD extract, and CBD-NLCs. The melting points for bulk CP and CBD were 48.4 °C and 67.0 °C, respectively, which corresponded to those reported in the literature [10,36]. No peaks for melting of the CBD were detected in the DSC thermograms of CBD-NLCs, suggesting that CBD was completely dissolved or presented in an amorphous state in the lipid matrix of the NLCs [23]. As shown in Table 3, the CBD-NLC formulations had melting temperatures ranging from 43 °C to 47 °C. The low melting temperature of the CBD-NLCs compared to that of bulk CP was due to the small particles in the nanosize range [37]. After production, the CBD-NLCs stabilized with TG450 showed a higher %CI compared to those stabilized with P188, suggesting that the type of surfactant affected the crystallization of the lipid matrix. Previous studies reported that surfactants could retard or accelerate the crystallization of the lipid matrices of SLNs and NLCs, depending on the chemical structure of the surfactant [37,38]. TG450 is a glyceryl diester of methylglucose (hydrophilic component) and stearic acid (hydrophobic component) [24], whereas P188 is a synthetic amphiphilic copolymer composed of a central hydrophobic poly(propylene oxide) (PPO) adjacent to two hydrophilic poly(ethylene oxide) (PEO) to form the triblock structure PEO-PPO-PEO [39]. As such, the long-chain hydrocarbons of TG450 embedded in the lipid matrices of the NLCs may have induced crystallization of the lipid matrices. Therefore, CBD-NLCs stabilized with TG450 showed higher %CI values than CBD-NLCs stabilized with P188.

To confirm the effects of surfactants on the crystallinity, the %CI values for all CBD-NLCs were determined after storage at 30 °C for 30 days. No CBD crystals were observed in any of the CBD-NLC formulations after 30 days of storage. However, it was found that the %CI values of the CBD-NLCs increased after storage, as shown in Table 3. The %CI values for CBD-NLCs stabilized with TG450 increased to a higher extent than those stabilized with P188. This result strongly confirmed that the surfactants affected the stabilities of the lipid matrices. Focusing on the effect of oil incorporation, CBD-NLC4 showed a lower %CI after 30 days than CBD-NLC2 and CBD-NLC3, indicating that the degree of disturbance of CP-matrix was modified by incorporation of oils with different structures.

### 3.6. X-ray Diffraction

**Figure 3:**
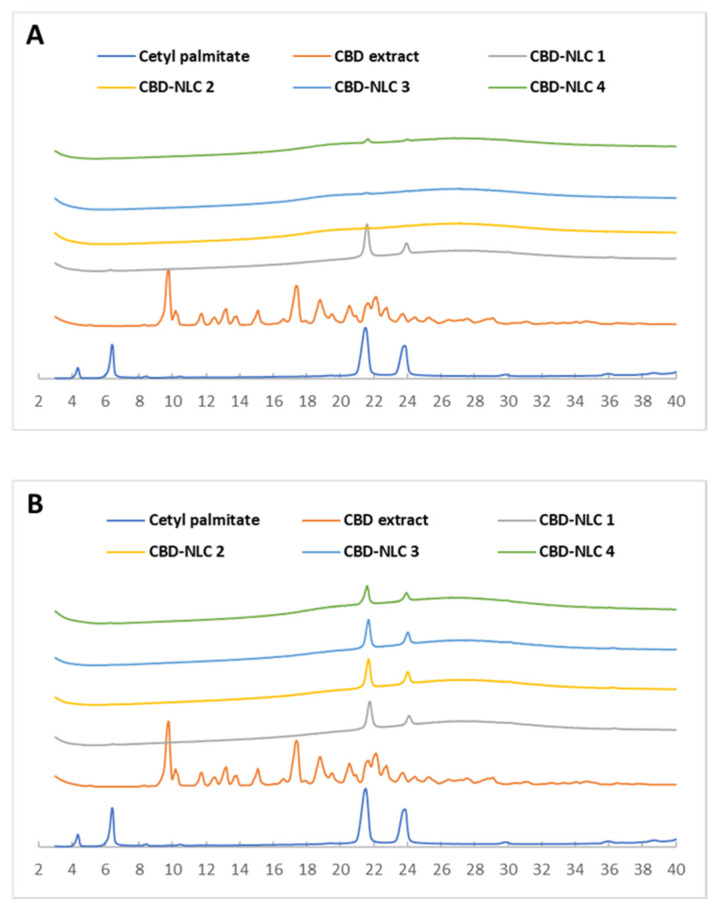
X-ray diffractograms of CP, CBD extract, and CBD-NLCs at the initial time (**A**) and after storage at 30 °C for 30 days (**B**).

X-ray diffraction was used to evaluate the polymorphism and changes in crystallinity of the CBD-NLCs. The X-ray diffractograms for CP, CBD extract, and CBD-NLCs at the initial time are shown in Figure 3A. CBD-NLCs exhibited d-spacings of 0.37–0.38 nm and 0.41–0.42 nm, indicating an orthorhombic subcell (β’-modification). The CBD extract diffractogram showed a crystalline structure with distinct peaks at d-spacings of 0.47 nm, 0.51 nm, 0.67 nm, and 0.90 nm. The X-ray diffractograms of all CBD-NLC formulations showed no characteristic peaks for CBD, confirming that the CBD has completely dissolved in the matrix of the lipid nanoparticles.

After 30 days of storage at 30 °C, the peak heights for the d-spacings of 0.37–0.38 nm and 0.41–0.42 nm had increased compared to those at the initial period (Figure 3B), suggesting an increase in the CP crystallinity. The results from X-ray diffraction were in accordance with those from the thermal analysis.

### 3.7. Photostability of CBD-NLCs

**Figure 4:**
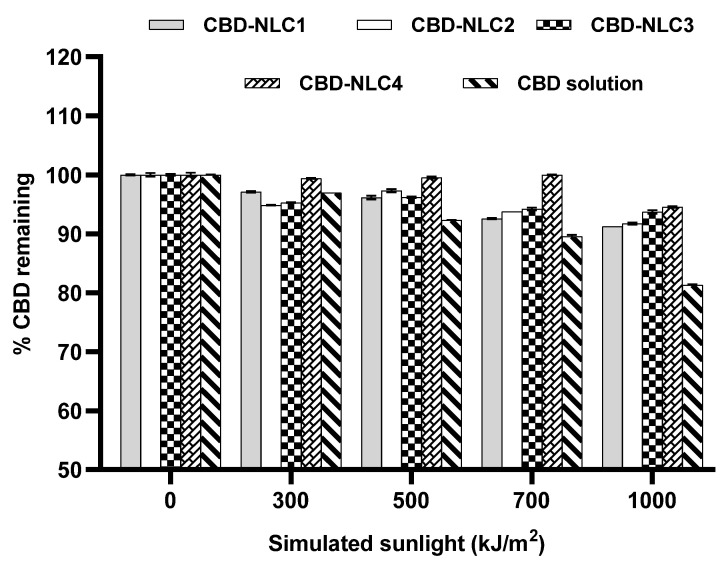
Percentage of CBD remaining after exposure to simulated sunlight at 0, 300, 500, 700, and 1000 kJ/m^2^.

In a previous study, it was reported that only 38% of commercially available products exhibited quantities within 10% of the label range. Moreover, CBD storage at a high temperature (37 °C) and with exposure to light accelerated degradation, with average values of up to 20% and 15%, respectively [12]. To evaluate the chemical stability of CBD-NLCs, the percentages of CBD remaining after exposure to simulated sunlight at 300, 500, 700, and 1000 kJ/m^2^ were analyzed by HPLC. Figure 4 shows the percentage of CBD remaining in the CBD extract in ethanol solution and in all CBD-NLC formulations. The amount of CBD remaining in the CBD extract solution was approximately 81% after exposure to 1000 kJ/m^2^, which was significantly lower than that in the CBD-NLCs (91–95%) (*p* < 0.05). The higher stability of CBD in the CBD-NLCs indicated stability improvement due to entrapment of the CBD in the lipid matrix of the NLCs. The percentages of CBD remaining for CBD-NLC1 and CBD-NLC2 were comparable (*p* > 0.05), implying that the surfactants used in this study did not affect the chemical stability of CBD entrapped in the NLCs.

In a comparison of all CBD-NLC formulations stabilized with the same surfactant (P188), CBD-NLC3 and CBD-NLC4 showed higher percentages of CBD remaining compared to CBD-NLC2 (*p* < 0.05). The high stability of CBD in CBD-NLC3 and CBD-NLC4 could be due to the difference in oil distributions in the CP matrices. MCT and OA containing CBD might be encapsulated in the inner structure of the CP matrix, while Transcutol^®^ P containing CBD might be distributed at the shell or on the surface of the CP matrix. The above results confirmed that the chemical stability of CBD in NLCs was improved by selecting suitable oils.

### 3.8. In Vitro Release

In vitro drug release was evaluated to observe the effects of formulation compositions on the release pattern of CBD-NLCs. The in vitro release studies of CBD from the CBD-NLCs were performed using vertical Franz diffusion cells. To maintain a sink condition throughout the in vitro release experiment, 2% Tween 20 was used as a receptor medium due to the high solubility of CBD in this medium.

**Figure 5:**
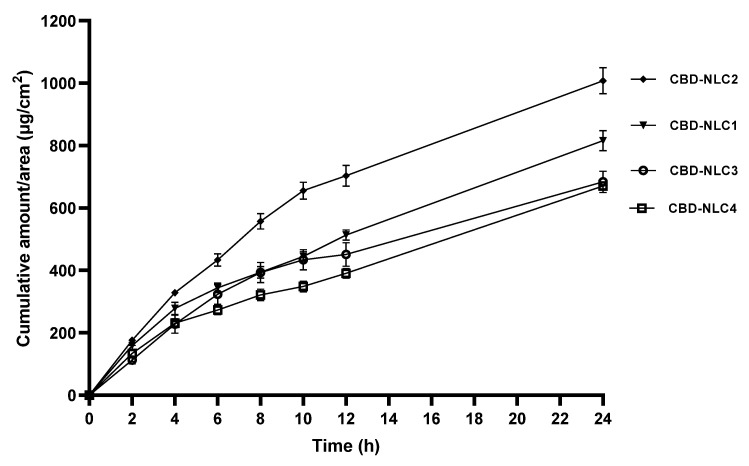
In vitro release of CBD-NLCs over a period of 24 h.

The CBD release profiles of CBD-NLCs are shown in Figure 5. In comparing the cumulative amounts of CBD released per area (μg/cm^2^) after 24 h, CBD-NLC2 showed the highest CBD release (*p* < 0.05), followed by CBD-NLC1, CBD-NLC3, and CBD-NLC4. The cumulative amounts of CBD released from CBD-NLC3 and CBD-NLC4 after 24 h were comparable (*p* > 0.05). Concerning the log P values of MCT, OA, and CBD, it was found that they were of the same magnitude (approx. 6–7), while the log P of Transcutol^®^ P is approx. 4. As a result, CBD would be compatible with MCT and OA. Consequently, CBD-NLCs prepared from MCT and OA showed slower release compared to that prepared from Transcutol^®^ P.

The release data of CBD from CBD-NLCs were fitted to the zero-order, first-order, Higuchi, and Korsmeyer–Peppas models, and the coefficients of determination (r^2^) for all formulations are shown in Table 4.

From Table 4, the release kinetics for CBD-NLCs were consistent with Higuchi’s model because the r^2^ values were higher than 0.95 for all formulations, indicating that CBD was slowly released from the CBD-NLC matrix at a rate proportional to the square root of time. As shown in the literature, the release kinetics of NLCs generally follow the Higuchi and Korsmeyer–Peppas models [24,40,41,42]. The Korsmeyer–Peppas model assumes that the release is controlled by diffusion [43], while the Higuchi model shows a direct relationship between the cumulative amount of drug released and the square root of time [44].

### 3.9. In Vitro Skin Permeation

**Figure 6:**
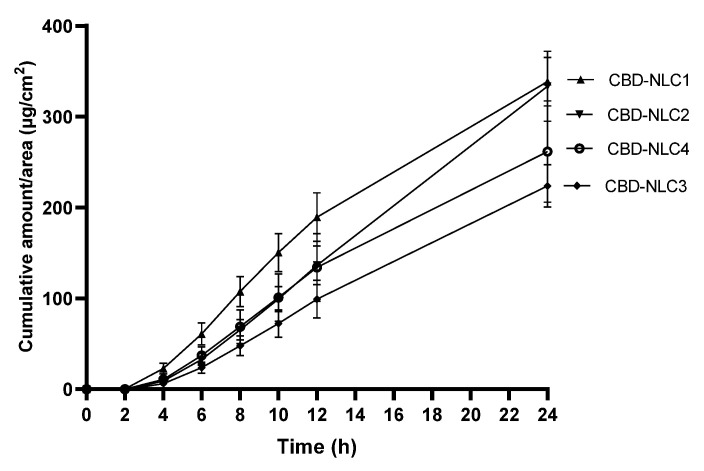
A cumulative amount of CBD permeation through the Strat-M^®^ membrane per area of CBD-NLCs over a period of 24 h.

The in vitro skin permeation of CBD-NLCs was evaluated using Strat-M^®^ as an alternative to a human skin model. The Strat-M^®^ membrane is designed to have structural and chemical characteristics similar to those of human skin. Strat-M^®^ contains lipids in a specific ratio similar to that of the human stratum corneum [44]. Previous studies showed good correlations between the skin permeability data for human skin and Strat-M^®^. In addition, Strat-M^®^ showed higher reproducibility from lot to lot compared to human skin. As a result, Strat-M^®^ can be used in pilot studies or as an alternative to animal or human skin for skin permeation studies [44,45,46]. The skin permeation profiles of CBD from CBD-NLCs are depicted in Figure 6. The cumulative amounts of CBD permeation after 24 h for CBD-NLC1 and CBD-NLC2 were not significantly different (*p* > 0.05) but were higher than those of CBD-NLC3 and CBD-NLC4. This could be explained by the higher CBD release rate from CBD-NLC1 and CBD-NLC2. Comparing CBD-NLC3 and CBD-NLC4, the cumulative amount of CBD permeation for CBD-NLC4 tended to be higher than that for CBD-NLC3. This might be due to the skin permeation enhancement of OA [9].

Table 5 displays the permeation fluxes at steady state (Flux_ss_) for the CBD-NLCs. CBD-NLC2 tended to show the highest flux, followed by CBD-NLC1, CBD-NLC4, and CBD-NLC3. Comparing the Flux_ss_ among formulations, it was found that Flux_ss_ of CBD-NLC2 was significantly higher than that of CBD-NLC3 (*p* < 0.05). From the above results, skin permeation of CBD can be adjusted by varying the types of surfactants and oils in the NLCs.

### 3.10. Cytotoxicity on Skin Cells

**Figure 7:**
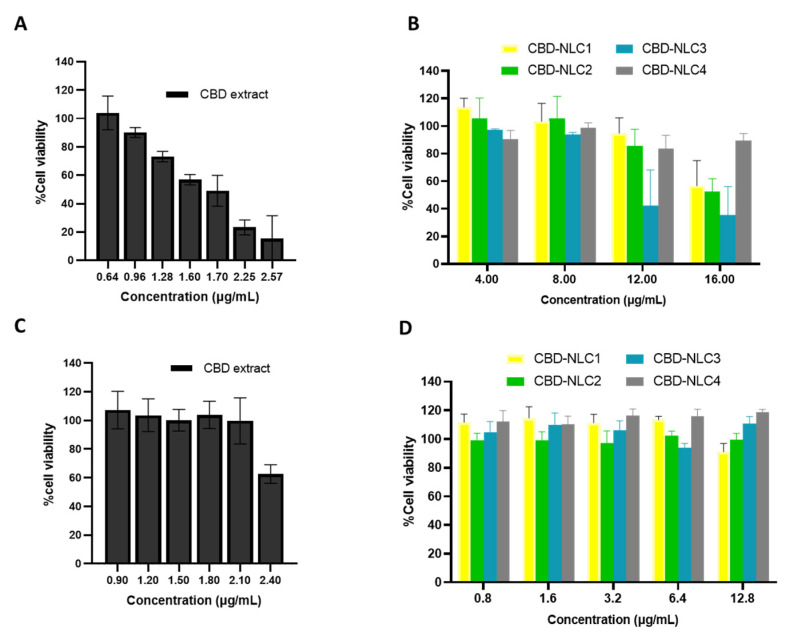
Cell viability after exposure to CBD extract and CBD-NLCs for 24 h of HaCaT cells (**A**,**B**) and HDF cells (**C**,**D**).

The viabilities of HaCaT cells treated with CBD extract and CBD-NLCs were determined after exposure for 24 h. The cytotoxicity of the CBD extract was determined within the concentration range of 0.64–2.57 µg/mL. Cell viabilities higher than 90% were observed after HaCaT cells were exposed to the CBD extract at concentrations less than or equal to 0.96 µg/mL (Figure 7A), which is equivalent to 0.84 µg/mL or 2.66 µM CBD.

Concerning the %cell viabilities of HaCaT cells after exposure to CBD-NLCs for 24 h, Figure 7B shows that the %cell viability for CBD-NLCs was higher than that for the CBD extract solution. For CBD-NLCs, cell viabilities higher than 90% were observed at concentrations less than or equal to 8 µg/mL, corresponding to 6.96 µg/mL or 22.13 µM CBD. These results showed that encapsulation of CBD in the lipid nanoparticles reduced the cytotoxicity of CBD to HaCaT cells.

Figure 7C,D shows the percentages of cell viability for the CBD extract solution and CBD-NLCs on HDFs after exposure for 24 h. The highest concentration of CBD extract that showed %cell viability higher than 90% was 2.1 µg/mL, which was equal to 1.83 µg/mL or 5.81 µM CBD. For the CBD-NLCs, cell viabilities higher than 90% were observed for concentrations less than or equal to 12.8 µg/mL, corresponding to 11.14 μg/mL or 35.41 µM. The cytotoxicities of CBD on HaCaT and HDF cells have been reported in several studies, while the reported CBD concentrations that were not toxic to HaCaT and HDF cells were different in each study [2,47,48,49,50,51]. For example, the study by Sangiovanni et al. indicated that CBD concentrations higher than 2.5 μM were toxic to HaCaT and HDF cells [47], whereas Petrosino et al. reported that treatment of HaCaT cells with 20 μM CBD did not cause toxicity to cells [51]. The different values for the cytotoxicity of CBD might be due to the different methodologies used in evaluating cell cytotoxicities.

Based on our results, it can be concluded that encapsulation of CBD in NLCs reduced the cytotoxicity of CBD on HaCaT and HDF cells, especially for CBD-NLC4. The lower cytotoxicity of CBD-NLC4 compared to the other CBD-NLC formulations might be due to the slow release of CBD from CBD-NLC4.

### 3.11. Anti-Inflammatory Effect in RAW264.7 Cell Macrophages

LPS is an extracellular component of Gram-negative bacteria, and it stimulates various cells, including monocytes and macrophages. When macrophages are exposed to LPS, inflammatory mediators such as IL-6 are released [52]. Recently, it was reported that treatment with CBD (0.02 mg/mL) significantly inhibited the LPS-induced levels of IL-6 and TNF-α produced in RAW264.7 cell macrophages [53]. In this study, the ability of CBD extract and CBD-NLCs to inhibit or reduce the production of IL-6 was studied in RAW264.7 cell macrophages by evaluating the concentrations of IL-6 in the culture supernatants. Firstly, the cell cytotoxicities of test samples were determined by an MTT assay using RAW264.7 cell macrophages at concentrations between 0.80 µg/mL and 12.8 µg/mL for CBD-NLCs and between 0.2 µg/mL and 3.6 µg/mL for the CBD extract. It was found that the concentrations of CBD extracts both in the CBD extract in 1% DMSO solution and in the CBD-NLCs at 0.8 µg/mL showed cell viabilities higher than 80%. According to the literature, a cell viability of 80% or higher determined by MTT assay is considered to indicate no cytotoxicity to the cells [54]. Therefore, the IL-6 inhibitory activities were studied at 0.8 µg/mL for the CBD extract solution and CBD-NLCs.

Table 6 shows the IL-6 inhibitory activities of diclofenac sodium, CBD extract solution, and CBD-NLCs. Treatment of RAW264.7 cell macrophages with LPS resulted in a significant increase in IL-6 production (573.4 pg/mL) compared to the negative control group (without LPS treatment) (3.4 pg/mL) (*p* < 0.05). The pretreatment of RAW264.7 cells with diclofenac sodium at a concentration of 1000 µM inhibited approximately 96% of the LPS-induced production of IL-6 in comparison with the positive control LPS-treated cells. CBD extract solution at a concentration of 0.8 µg/mL inhibited IL-6 production induced by LPS by 15.7%. For CBD-NLCs, the percentages for inhibition of IL-6 production were in the range 5–37%. The extents to which CBD-NLC1 and CBD-NLC2 inhibited LPS-induced IL-6 production were comparable (*p* > 0.05) and in the range 5–6%, which were lower than that of CBD extract solution. The lower IL-6 inhibitory activities of CBD-NLC1 and CBD-NLC2 were likely due to the encapsulation of CBD extract in the NLCs, resulting in the low concentration of CBD extract in the medium as compared to free CBD extract which was used as a control. Interestingly, CBD-NLC4 (37.0%) and CBD-NLC3 (24.3%) showed higher inhibition percentages than the CBD extract solution even though they showed lower CBD release. The higher IL-6 inhibitory activities of CBD-NLC3 and CBD-NLC4 might be due to the anti-inflammatory effects of the oil composition (MCT and OA). OA, an unsaturated fatty acid, was reported to exhibit anti-inflammatory activity by decreasing IL-6 levels in either LPS-induced THP-1-derived macrophages and monocytes [55] or in Murin RAW264.7 macrophages [52]. Yu et al. reported that MCT inhibited the production of proinflammatory cytokines, including IL-6 [56]. In addition, OA is known to interact with saturated lipid molecules and alter the structures, fluidities, and permeabilities of lipid membranes [57]. As a result, encapsulation of the CBD extract into NLCs containing OA and MCT showed a higher anti-inflammatory effect than CBD-NLC1 or CBD-NLC2.

## 4. Conclusions

In this study, CBD-NLCs were successfully developed with high E.E. of 100%. The type of surfactants and oils affected the physicochemical properties of the CBD-NLCs, such as particle sizes, zeta potentials, and % crystallinities. In addition, the release and permeation of CBD depended on the composition of CBD-NLCs. The photostability of the CBD extract was enhanced by encapsulation in the NLCs. Based on cytotoxicity studies on HaCaT and HDF cells, it was found that encapsulation of CBD in NLCs reduced the cytotoxicity of CBD. In the anti-inflammatory activity study, CBD-NLCs containing MCT and OA effectively enhanced the anti-inflammatory activities of CBD in LPS-induced RAW264.7 cell macrophages.

## Figures and Tables

**Table 1:** Compositions of CBD-NLC formulations (%*w*/*w*).

Compositions	CBD-NLC1	CBD-NLC2	CBD-NLC3	CBD-NLC4
CP	8	8	8	8
CBD extract	1	1	1	1
TG450	1.8	-	-	-
P188	-	2.5	2.5	2.5
Transcutol^®^ P	2	2	-	-
MCT	-	-	2	-
OA	-	-	-	2
Unigerm G2	1	1	1	1
Water q.s. to	100	100	100	100

**Table 2:** Particle size, PDI, and zeta potential of CBD-NLCs.

Formulation	Particle Size (nm)	PDI	Zeta Potential (mV)
CBD-NLC1	165.4 ± 0.8	0.009 ± 0.011	−57.0 ± 1.7
CBD-NLC2	163.2 ± 1.2	0.073 ± 0.009	−29.0 ± 0.5
CBD-NLC3	175.5 ± 1.9	0.087 ± 0.023	−31.1 ± 0.8
CBD-NLC4	166.9 ± 1.5	0.061 ± 0.020	−39.8 ± 1.0

**Table 3:** DSC parameters of bulk CP, CBD extract, and CBD-NLCs at an initial time and after 30 days of storage at 30 °C.

Formulation	Condition	Onset (°C)	Endset (°C)	Melting Point (°C)	%CI
CBD extract	Initial	65.7	68.0	67.0	-
CP	Initial	44.2	50.3	48.4	100.00
CBD-NLC1	Initial	35.0	47.5	43.3	9.6
	30 days	41.5	47.5	46.0	49.6
CBD-NLC2	Initial	44.6	47.9	46.9	2.1
	30 days	43.8	47.8	46.7	37.6
CBD-NLC3	Initial	44.1	48.0	46.2	2.6
	30 days	44.4	47.9	46.8	39.6
CBD-NLC4	Initial	42.3	48.4	46.0	0.3
	30 days	43.0	47.0	45.6	17.7

**Table 4:** The coefficient of determination (r^2^) of release kinetics of CBD from CBD-NLCs fitted to zero-order, first-order, Higuchi’s model, and Korsmeyer–Peppas.

Formulation	Zero-Order	First-Order	Higuchi’s Model	Korsmeyer–Peppas
CBD-NLC1	0.9503	0.9630	0.9813	0.9441
CBD-NLC2	0.9128	0.9326	0.9820	0.9761
CBD-NLC3	0.9439	0.9186	0.9815	0.9800
CBD-NLC4	0.9549	0.9640	0.9720	0.9246

**Table 5:** Flux of skin permeation at a steady state (µg/cm^2^/h) of CBD-NLCs.

Formulation	Flux_ss_ (µg/cm^2^/h)
CBD-NLC1	15.56 ± 1.22 ^a,b^
CBD-NLC2	16.47 ± 1.81 ^a^
CBD-NLC3	11.00 ± 1.02 ^b^
CBD-NLC4	12.52 ± 2.71 ^a,b^

The different letters indicate a statistically significant difference among formulations at a significant level of 0.05 (*p* < 0.05).

**Table 6:** IL-6 level and %IL-6 inhibition of CBD extract and CBD-NLCs in RAW264.7 cell macrophages.

Formulation	IL-6 Level (pg/mL)	%IL-6 Inhibition over Positive Control
Negative control	3.4 ± 1.8	-
Positive control	573.4 ± 4.0	0.00
CBD extract	483.1 ± 16.3	15.7 ± 2.8
CBD-NLC1	540.0 ± 8.4	5.8 ± 1.5
CBD-NLC2	540.7 ± 9.0	5.7 ± 1.6
CBD-NLC3	433.98 ± 38.7	24.3 ± 6.7
CBD-NLC4	361.4 ± 4.2	37.0 ± 0.7

## Data Availability

Data are contained within the article.
